# Dietary iron intake and its impact on osteopenia/osteoporosis

**DOI:** 10.1186/s12902-023-01389-0

**Published:** 2023-07-18

**Authors:** Xin Liu, Jingjing An

**Affiliations:** 1 Operating room, West China hospital, Sichuan University, Chengdu, 610000 Sichuan Province China; 2 West China School of Nursing, Sichuan University, Chengdu, 610000 Sichuan Province China

**Keywords:** Dietary iron intake, Osteopenia, Osteoporosis, Bone mineral density

## Abstract

**Background:**

Osteoporosis is a prevalent condition characterized by low bone density and increased risk of fractures, resulting in a significant healthcare burden. Previous research has suggested that serum ferritin levels may be related to the risk of developing osteoporosis. The aim of this study was to investigate the relationship between dietary iron intake and the development of osteoporosis.

**Methods:**

Using data from the National Health and Nutrition Examination Survey (NHANES) conducted between 2005 and 2018, a total of 11,690 adults aged over 20 were evaluated. Bone mineral density (BMD) measurements of the femoral neck and lumbar spine were used to assess osteoporosis and osteopenia. Dietary iron intake was determined using food intake interviews and the Food and Nutrient Database for Dietary Studies. Logistic regression models were applied to investigate the association between dietary iron consumption and osteopenia and osteoporosis.

**Results:**

After adjusting for sociodemographic factors, compared with those who had the first quartile (Q1) of dietary iron intake, the odds ratio (OR) for osteopenia across the quartiles of dietary iron intake levels was 0.88 (95%CI: 0.79–0.98), 0.80 (95%CI: 0.72–0.89), and 0.74 (95%CI: 0.67–0.83) for Q2, Q3, and Q4, respectively. And the OR for osteoporosis across the quartiles of dietary iron intake levels was 1.00, 0.77 (95%CI: 0.50–1.19), 0.54 (95%CI: 0.34–0.89), and 0.83 (95%CI: 0.54–1.29) for Q1, Q2, Q3, and Q4, respectively. Notably, the observed association was significant among females but not males.

**Conclusion:**

The risk of osteopenia/osteoporosis in females decreases with a moderate increase in dietary iron consumption. For females to preserve bone health, moderately increasing their dietary iron intake without overindulging should be seen as a key approach. Our study provides useful insights for developing dietary strategies to prevent and manage osteoporosis in vulnerable populations.

## Background

Osteoporosis, a systemic bone disease characterized by osteopenia and bone microstructure deterioration, has emerged as a significant public health concern in the middle-aged and elderly population due to the aging of the population [1, 2]. The prevalence of osteoporosis increases with age, affecting 36% of Chinese people over the age of 60, with higher rates among women (49%) than men (23%) [3]. Osteoporosis-related fractures are common among people aged over 50 years old, with 50% of females and 20% of males suffering from such fractures [3]. These fractures have a substantial impact on patients’ quality of life, resulting in disability and even mortality. One-year mortality rates following hip fractures have been reported to be as high as 20%, with approximately 50% of patients becoming disabled [4]. The economic burden of fractures is also substantial, with medical expenditures for fractures in China totaling $10.2 billion in 2010 and expected to reach $275 billion by 2050 [5].

Diet plays a crucial role in preserving bone mass and maintaining bone health throughout life, as the bone is an active and dynamic tissue that requires adequate nutrients during remodeling and mineralization [6, 7]. Dietary nutrients, such as protein, vitamin D, and calcium, have been shown to affect bone remodeling regulation [8], while micronutrients such as iron and zinc are associated with bone homeostasis [9]. Iron deficiency has been identified as an important factor affecting bone health, with plasma iron levels found to be associated with the risk of osteoporosis in a U-shaped exposure-response relationship [10]. In addition to plasma iron levels, studies have also demonstrated that both iron excess and a lack of iron in the diet can harm bone mass and mineral content [11–13]. Iron deficiency may impact bone health by affecting vitamin D metabolism and collagen synthesis, and iron overload suppresses osteoblast proliferation and differentiation while promoting osteoclast differentiation [14, 15].

Despite the potential importance of iron intake in maintaining bone health, research on the association between dietary iron consumption and adult osteoporosis is limited. Therefore, the aim of the current study is to investigate the relationship between dietary iron intake and osteopenia and osteoporosis in adults.

## Methods

### Study design and population

In the US, NHANES is an ongoing health examination study and nutritional status survey that includes adults and children. Initiating from 1998, five data components are included in the project: demographic, dietary, examination, laboratory, and questionnaire. Participants were interviewed at home, and examinations and laboratory tests were conducted at mobile examination centers. The continuous NHANES survey design can be found at http://www.cdc.gov/nchs/nhanes/index.htm. Informed consent was obtained from all participants before data collection by the ethics review board of the National Center for Health Statistics. Seven survey cycles were used in the present study (2005–2006, 2007–2008, 2009–2010, 2011–2012, 2013–2014, 2015–2016, 2017–2018). Collectively, there were 70,190 participants in the pooled cycles. Participants with missing values for dietary iron intake and/or osteopenia/osteoporosis, or aged < 20 years, were removed from the sample pool. The final sample for multiple cross-sectional analyses comprised 11,690 participants.

### Assessment of osteopenia and osteoporosis

The osteopenia was assessed via bone mineral density (BMD) measurement for the femoral neck and lumbar spine. Firstly, all data for BMD were standardized for eliminating bias from units. Secondly, osteopenia was defined as BMD of femoral neck lower than − 1 and greater than − 2.5, and BMD of lumbar spine lower than − 1 and great than − 2.5. Thirdly, osteoporosis was detected while BMD of the femoral neck or lumbar spine was lower than − 2.5 [16].

### Assessment of dietary iron intake

NHANES interviewed food intake on two non-consecutive days, the first in person and the second by telephone. Due to the much missing in the second-wave interview, we used the first-wave food intake in this analysis, and an estimation of dietary iron-nutrient was made using the Food and Nutrient Database for Dietary Studies published by the United States Department of Agriculture [17].

### Covariates

The confirmed age was recorded at the time of screening. Sex was dichotomized into male and female. Covariates definition refers to the previous study [18]. Races were grouped into Mexican American, non-Hispanic Black, non-Hispanic White, other Hispanic, and other race. Educational attainment was divided as less than high school, high school, and above high school. The ratio of family income to poverty was an income index estimated household socioeconomic status, which was graded as < 1.5, 1.5–3.5, or > 3.5 [19]. Alcohol intake was defined as mild, moderate, and heavy [20, 21]. Heavy alcohol use was defined as ≥ 3 drinks per day for females or ≥ 4 drinks per day for males, or binge drinking on five or more days per month. Moderate alcohol use was defined as ≥ 2 drinks per day for females and ≥ 3 drinks per day for males, or binge drinking ≥ 2 days per month. Mild alcohol use was regarded as others. Smoking status was defined as never, former, and current. Never smokers were those who smoked < 100 cigarettes in their lifetime; former smokers smoked > 100 cigarettes in their lifetime but currently did not smoke at all, and current smokers smoked > 100 cigarettes in their lifetime and currently smoked some days or every day. Cardiovascular disease (CVD) was defined as a report of CVD diagnosed by a physician, or taking anti-CVD medicine. Physical activity level was calculated using the metabolic equivalent (MET, min/week) [22]. The body mass index (BMI) was classified as underweight (< 20 kg/m2), normal (≥ 20 kg/m2 to < 25 kg/m2), overweight (≥ 25 kg/m2 to < 30 kg/m2), and obese (≥ 30 kg/m2) [23]. Diabetes mellitus was defined as reporting a diabetes diagnosis, glycohemoglobin HbA1c (%) > 6.5, or fasting glucose (mmol/L) ≥ 7.0, random blood glucose (mmol/L) ≥ 11.1, two-hour OGTT blood glucose (mmol/L) ≥ 11.1, or use of diabetes medication or insulin [24].

### Statistical analysis

The mean dietary iron intake was grouped into quartiles from the lowest (first quartile, Q1) to the highest (fourth quartile, Q4). We summarized continuous variables by means and standard deviations and categorical variables with numbers and proportions. Analyzing data for normally distributed data using one-way ANOVA and categorical data using Chi-square tests determined differences among quartiles in dietary iron intake. To account for oversampling and non-responses, a seven-cycle sample weight (2005–2018) was used. Using tutorials on the NHANES website, we defined primary sample units and strata based on complex multistage probability sampling. An analysis of the associations between dietary iron intake quartiles and osteopenia or osteoporosis was conducted using three logistic regression models. Model 1 was no covariate; Model 2 was adjusted for age, sex, race, education, the ratio of family income to poverty, educational level, alcohol intake, smoking status, and MET. Model 3 was further adjusted for cardiovascular disease, BMI, and diabetes. Odds ratio (OR) and 95% confidence interval (CI) were used to assess the strength of the association.

Additionally, some additional analyses were conducted. As a first step, dietary iron intake was included as continuous variables in three logistic models with osteopenia and osteoporosis, not as quartiles. The second step was to perform interactive analyses with osteopenia and osteoporosis stratified by sex, races, education, alcohol intake, smoking status, the ratio of family poverty, CVD, and diabetes. Statistical analyses were conducted using STATA software (Version 17, Stata Corporation). Two-tailed *P* < 0.05 was defined as the significant threshold.

## Results

There were 11,690 participants included in the final analysis across the quartiles of dietary iron intake levels, as shown in Table 1. Participants in the Q4 group tended to be younger, male, non-Hispanic White, above high school attainment, mild alcohol consumption, never smokers, overweight, with highest MET levels, the ratio of family income to poverty > 3.5, no diabetes, and cardiovascular disease, no osteopenia, and osteoporosis. There was a significant difference among the dietary iron intake quartiles in terms of age, sex, education, race, alcohol intake, smoking status, BMI, the MET, the ratio of family income to poverty, diabetes, osteopenia and osteoporosis except for CVD (*P* < 0.05).

**Table 1:** Characteristic distribution of the participants in NHANES 2005–2018 across dietary iron intake

Variables		Q1	Q2	Q3	Q4	*P*-value
		N = 2,924	N = 2,922	N = 2,927	N = 2,917	
Age		63.6 (10.5)	63.2 (10.6)	62.5 (10.6)	62.0 (10.9)	< 0.001
Sex	Female	1,789 (61.2%)	1,526 (52.2%)	1,271 (43.4%)	889 (30.5%)	< 0.001
	Male	1,135 (38.8%)	1,396 (47.8%)	1,656 (56.6%)	2,028 (69.5%)	
Educational attainment	Above high school	1,571 (53.9%)	1,683 (57.7%)	1,727 (59.1%)	1,793 (61.5%)	< 0.001
	High school	884 (30.3%)	845 (28.9%)	830 (28.4%)	800 (27.5%)	
	Less than high school	461 (15.8%)	391 (13.4%)	367 (12.6%)	321 (11.0%)	
Sex	Female	1,789 (61.2%)	1,526 (52.2%)	1,271 (43.4%)	889 (30.5%)	< 0.001
	Male	1,135 (38.8%)	1,396 (47.8%)	1,656 (56.6%)	2,028 (69.5%)	
Races	Mexican American	372 (12.7%)	428 (14.6%)	441 (15.1%)	411 (14.1%)	< 0.001
	Non-Hispanic Black	760 (26.0%)	570 (19.5%)	526 (18.0%)	457 (15.7%)	
	Non-Hispanic White	1,259 (43.1%)	1,406 (48.1%)	1,486 (50.8%)	1,621 (55.6%)	
	Other Hispanic	322 (11.0%)	267 ( 9.1%)	241 ( 8.2%)	225 ( 7.7%)	
	Other Race - Including Multi-Racial	211 ( 7.2%)	251 ( 8.6%)	233 ( 8.0%)	203 ( 7.0%)	
Alcohol intake	Heavy	281 (17.6%)	301 (17.4%)	290 (16.4%)	314 (17.1%)	< 0.001
	Mild	912 (57.3%)	1,008 (58.4%)	1,102 (62.2%)	1,171 (63.7%)	
	Moderate	400 (25.1%)	417 (24.2%)	379 (21.4%)	352 (19.2%)	
Smoking status	Former	836 (28.6%)	973 (33.3%)	1,007 (34.4%)	1,035 (35.5%)	< 0.001
	Never	1,510 (51.7%)	1,439 (49.2%)	1,462 (50.0%)	1,365 (46.8%)	
	Now	576 (19.7%)	510 (17.5%)	457 (15.6%)	517 (17.7%)	
BMI	0–20	135 ( 4.6%)	112 ( 3.9%)	83 ( 2.8%)	102 ( 3.5%)	0.005
	20–25	654 (22.5%)	628 (21.6%)	649 (22.3%)	689 (23.7%)	
	25–30	1,057 (36.4%)	1,132 (39.0%)	1,137 (39.0%)	1,139 (39.2%)	
	30-	1,060 (36.5%)	1,034 (35.6%)	1,046 (35.9%)	979 (33.7%)	
Physical activity, (MET, per week)		3437.6 (5656.9)	3577.1 (5792.3)	3564.8 (5279.1)	3951.5 (5924.2)	0.023
The ratio of family poverty	0-1.5	1,011 (38.3%)	830 (31.3%)	774 (29.1%)	759 (28.2%)	< 0.001
	1.5–3.5	913 (34.6%)	915 (34.5%)	896 (33.7%)	884 (32.8%)	
	3.5-	713 (27.0%)	911 (34.3%)	987 (37.1%)	1,051 (39.0%)	
Diabetes	DM	847 (29.0%)	758 (25.9%)	750 (25.6%)	690 (23.7%)	< 0.001
	IFG	151 ( 5.2%)	136 ( 4.7%)	168 ( 5.7%)	192 ( 6.6%)	
	IGT	137 ( 4.7%)	141 ( 4.8%)	140 ( 4.8%)	140 ( 4.8%)	
	No	1,789 (61.2%)	1,887 (64.6%)	1,869 (63.9%)	1,895 (65.0%)	
CVD	No	2,396 (81.9%)	2,413 (82.6%)	2,452 (83.8%)	2,432 (83.4%)	0.24
	Yes	528 (18.1%)	508 (17.4%)	474 (16.2%)	485 (16.6%)	
Osteopenia	No	1,449 (54.1%)	1,555 (57.2%)	1,651 (59.7%)	1,685 (61.3%)	< 0.001
	Yes	1,230 (45.9%)	1,165 (42.8%)	1,116 (40.3%)	1,063 (38.7%)	
Osteoporosis	No	1,449 (85.5%)	1,555 (88.5%)	1,651 (91.2%)	1,685 (90.9%)	< 0.001
	Yes	245 (14.5%)	202 (11.5%)	160 ( 8.8%)	169 ( 9.1%)	

Note. BMI: body mass index; DM: diabetes mellitus; MET: metabolic equivalent; Q: quartile; CVD: Cardiovascular disease; IFG: Impaired Fasting Glycaemia; IGT: Impaired Glucose Tolerance

All values are presented as number (%) for categorical variables or mean (standard deviation) for continuous variables

High dietary iron intake levels were negatively associated with osteopenia in the null model, and the OR for osteopenia across the quartiles of dietary iron intake levels was 1.00, 0.88 (95%CI: 0.79–0.98), 0.80 (95%CI: 0.72–0.89), and 0.74 (95%CI: 0.67–0.83) for Q1, Q2, Q3, and Q4, respectively. After adjusting for demographic variables, the association was not significantly changed, and the OR of the dietary iron intake level quartiles was 1.00, 0.77 (95% CI: 0.64–0.93), 0.74 (95% CI: 0.61–0.89), and 0.79 (95% CI: 0.65–0.95) for Q1, Q2, Q3, and Q4, respectively. In the full mode, after further adjusting for CVD, BMI, and diabetes, the OR for osteopenia across the dietary iron intake level quartiles were 1.00, 0.78 (95%CI: 0.65–0.95), 0.75 (95%CI: 0.62–0.91), and 0.78 (95%CI: 0.65–0.95) for Q1, Q2, Q3, and Q4, respectively (Table 2). In the full model, the mean dietary iron intake was associated with a lower risk of osteopenia (OR 0.99, 95%CI: 0.98–0.99). A significant linear association was found between the quartiles of dietary iron intake levels and osteopenia (*P* for trend < 0.001) (Table 2). No significant moderating effect was found in the subgroup analysis stratified by sex, race, education, alcohol intake, smoking status, the ratio of family income to poverty, CVD, and diabetes (*P* > 0.05) (Table 3).

**Table 2:** Association between dietary iron intake and osteopenia among U.S. adults in NHANES 2005–2018

	Crude model	Model 1	Model 2
	OR (95% CI)	OR (95% CI)	OR (95% CI)
Continuous	0.99 (0.98–0.99)	0.99 (0.98–0.99)	0.99 (0.98–0.99)
Quartile			
Q1	1	1	1
Q2	0.88 (0.79–0.98)	0.77 (0.64–0.93)	0.78 (0.65–0.95)
Q3	0.80 (0.72–0.89)	0.74 (0.61–0.89)	0.75 (0.62–0.91)
Q4	0.74 (0.67–0.83)	0.79 (0.65–0.95)	0.78 (0.65–0.95)
P for trend	< 0.001	< 0.001	< 0.05

Crude model: no adjustment

Model 1: adjusted sex, races, education, alcohol intake, smoking status, the ratio of family poverty, and MET

Model 2: further adjusted CVD, BMI, and diabetes

**Table 3:** Subgroup analysis of association between dietary iron intake and osteopenia among U.S. adults in NHANES 2005–2018

	Quartiles of iron							
Variables	Q1	Q2		Q3		Q4		P for interaction
Sex								0.557
Female	1.00	0.74	(0.55–0.98)	0.63	(0.47–0.84)	0.70	(0.50–0.98)	
Male	1.00	0.81	(0.60–1.09)	0.81	(0.61–1.09)	0.86	(0.65–1.14)	
Races								0.747
Mexican American	1.00	0.72	(0.39–1.33)	0.59	(0.31–1.11)	0.99	(0.53–1.86)	
Non-Hispanic Black	1.00	0.69	(0.41–1.13)	0.76	(0.45–1.30)	0.65	(0.37–1.12)	
Non-Hispanic White	1.00	0.78	(0.59–1.03)	0.73	(0.56–0.95)	0.81	(0.62–1.06)	
Other Hispanic	1.00	0.44	(0.22–0.91)	0.50	(0.25–1.02)	0.54	(0.25–1.13)	
Other Race - Including Multi-Racial	1.00	1.64	(0.73–3.68)	1.32	(0.59–2.93)	0.90	(0.39–2.07)	
Education								0.371
Above high school	1.00	0.79	(0.62–1.01)	0.74	(0.58–0.94)	0.83	(0.65–1.07)	
High school	1.00	0.54	(0.36–0.83)	0.64	(0.42–0.97)	0.63	(0.41–0.96)	
Less than high school	1.00	1.48	(0.67–3.26)	0.84	(0.36–1.97)	1.16	(0.51–2.61)	
Alcohol intake								0.146
Heavy	1.00	0.85	(0.53–1.37)	0.42	(0.25–0.70)	0.62	(0.38–1.02)	
Mild	1.00	0.76	(0.58–1.00)	0.77	(0.59–1.00)	0.82	(0.63–1.08)	
Moderate	1.00	0.72	(0.48–1.07)	0.90	(0.60–1.36)	0.80	(0.52–1.22)	
Smoking status								0.478
Former	1.00	0.81	(0.56–1.15)	0.85	(0.60–1.20)	0.82	(0.57–1.18)	
Never	1.00	0.71	(0.52–0.96)	0.60	(0.45–0.82)	0.69	(0.51–0.94)	
Now	1.00	0.78	(0.49–1.22)	0.74	(0.46–1.20)	1.03	(0.65–1.62)	
The ratio of family income to poverty								0.640
0-1.5	1.00	0.72	(0.49–1.06)	0.62	(0.41–0.93)	0.94	(0.62–1.43)	
1.5–3.5	1.00	0.74	(0.51–1.07)	0.82	(0.57–1.17)	0.79	(0.55–1.13)	
3.5-	1.00	0.79	(0.58–1.09)	0.70	(0.51–0.96)	0.72	(0.52–1.00)	
CVD								0.841
No	1.00	0.78	(0.63–0.97)	0.72	(0.58–0.89)	0.82	(0.65–1.02)	
Yes	1.00	0.68	(0.39–1.20)	0.73	(0.41–1.31)	0.66	(0.37–1.16)	
DM								0.171
No	1.00	0.73	(0.58–0.92)	0.64	(0.51–0.81)	0.76	(0.60–0.95)	
Yes	1.00	0.94	(0.60–1.47)	1.13	(0.72–1.75)	0.93	(0.59–1.45)	

Note. Adjusted for age, sex, races, education, alcohol intake, smoking status, the ratio of family income poverty, MET, cardiovascular disease, BMI, diabetes. Q1-Q4: Quartile 1 – Quartile 4. CVD: Cardiovascular disease, BMI: body mass index; DM: diabetes mellitus; MET: metabolic equivalent

**Table 4:** Association between dietary iron intake and osteoporosis among U.S. adults in NHANES 2005–2018

	Crude model	Model 1	Model 2
	OR(95% CI)	OR(95% CI)	OR(95% CI)
Continuous	0.97 (0.96–0.98)	1.00 (0.98–1.02)	0.99 (0.97–1.01)
Quartile			
Q1	1	1	1
Q2	0.77 (0.63–0.94)	0.73 (0.48–1.11)	0.77 (0.50–1.19)
Q3	0.57 (0.46–0.71)	0.54 (0.35–0.85)	0.54 (0.34–0.85)
Q4	0.59 (0.48–0.73)	0.89 (0.58–1.36)	0.83 (0.54–1.29)
P for trend	< 0.05	0.372	0.215

Crude model: no adjustment

Model 1: adjusted sex, races, education, alcohol intake, smoking status, the ratio of family poverty, and MET

Model 2: further adjusted CVD, BMI, and diabetes

**Table 5:** Subgroup analysis of association between dietary iron intake and osteoporosis among U.S. adults in NHANES 2005–2018

	Quartiles of iron							
Variables	Q1	Q2		Q3		Q4		*P* for interaction
Sex								0.767
Female	1.00	0.81	(0.47–1.40)	0.38	(0.20–0.72)	0.60	(0.31–1.18)	
Male	1.00	0.75	(0.32–1.77)	0.61	(0.26–1.44)	0.93	(0.43–1.98)	
Races								0.851
Mexican American	1.00	0.94	(0.19–4.72)	0.40	(0.07–2.36)	2.29	(0.41–12.72)	
Non-Hispanic Black	1.00	0.81	(0.20–3.18)	0.22	(0.03–1.93)	0.59	(0.13–2.64)	
Non-Hispanic White	1.00	0.89	(0.50–1.60)	0.53	(0.28–1.01)	0.71	(0.38–1.33)	
Other Hispanic	1.00	0.13	(0.02–0.90)	0.08	(0.01–0.95)	0.27	(0.04–1.79)	
Other Race - Including Multi-Racial	1.00	1.50	(0.22–10.37)	1.67	(0.25–11.16)	0.92	(0.10–8.25)	
Education								0.817
Above high school	1.00	0.75	(0.44–1.26)	0.51	(0.29–0.90)	0.63	(0.36–1.13)	
High school	1.00	1.01	(0.36–2.79)	0.28	(0.07–1.06)	1.03	(0.37–2.90)	
Less than high school	1.00	0.60	(0.07–5.14)	0.55	(0.05–6.14)	1.30	(0.12–13.59)	
Alcohol intake								0.513
Heavy	1.00	1.68	(0.51–5.60)	0.51	(0.12–2.12)	1.74	(0.50–6.05)	
Mild	1.00	0.77	(0.42–1.43)	0.45	(0.23–0.88)	0.71	(0.37–1.37)	
Moderate	1.00	0.67	(0.29–1.56)	0.54	(0.21–1.38)	0.46	(0.16–1.28)	
Smoking status								0.693
Former	1.00	0.66	(0.30–1.49)	0.38	(0.15–0.93)	0.50	(0.21–1.20)	
Never	1.00	0.83	(0.42–1.66)	0.50	(0.24–1.05)	0.71	(0.33–1.52)	
Now	1.00	0.87	(0.35–2.12)	0.53	(0.17–1.67)	1.28	(0.51–3.22)	
The ratio of family poverty								0.026
0-1.5	1.00	0.64	(0.26–1.56)	0.38	(0.13–1.11)	1.32	(0.52–3.34)	
1.5–3.5	1.00	0.64	(0.29–1.40)	0.32	(0.13–0.79)	0.98	(0.46–2.06)	
3.5-	1.00	1.13	(0.53–2.41)	0.78	(0.35–1.74)	0.25	(0.08–0.80)	
CVD								0.425
No	1.00	0.90	(0.56–1.45)	0.49	(0.29–0.84)	0.81	(0.48–1.35)	
Yes	1.00	0.33	(0.06–1.78)	0.91	(0.19–4.44)	0.91	(0.22–3.69)	
Diabetes								0.479
No	1.00	0.74	(0.45–1.22)	0.41	(0.23–0.72)	0.77	(0.46–1.29)	
Yes	1.00	1.59	(0.50–5.12)	1.46	(0.44–4.82)	1.09	(0.28–4.30)	

Note. Adjusted for age, sex, races, education, alcohol intake, smoking status, the ratio of family income poverty, MET, cardiovascular disease, BMI, diabetes. Q1-Q4: Quartile 1 – Quartile 4. CVD: Cardiovascular disease, BMI: body mass index; DM: diabetes mellitus; MET: metabolic equivalent

High dietary iron intake levels had a close reverse relation with osteoporosis in quartile 3 not quartile 4 in the full model, and the OR for osteoporosis across the quartiles of dietary iron intake levels was 1.00, 0.77 (95%CI: 0.50–1.19), 0.54 (95%CI: 0.34–0.89), and 0.83 (95%CI: 0.54–1.29) for Q1, Q2, Q3, and Q4, respectively. Stratification analyses by gender showed that the association was significant in women, but not men. Additionally, the mean dietary iron intake was not associated with the occurrence of osteoporosis in the final model (OR 0.99, 95%CI: 0.97–1.01) (Table 4). There was no significant between-group difference in dietary iron intake quartiles in the subgroup analysis stratified by races, education, alcohol intake, smoking status, the ratio of family income to poverty, CVD, and diabetes (*P* > 0.05) (Table 5).

## Discussion

In this national prospective cohort study, we found a significant association between dietary iron intake and the risk of osteopenia/osteoporosis. Specifically, we observed a U-shaped relationship between dietary iron intake and decreased osteoporosis risk, which was independent of sociodemographic factors. Our findings highlight the importance of considering dietary iron intake as a potential preventive factor for osteopenia/osteoporosis .

The effects of diet on bone cells can be divided into pro-anabolic effects, which promote bone formation, and anti-catabolic effects, which inhibit bone resorption [25, 26].Pro-anabolic effects can be achieved through the intake of nutrients that support bone formation. For example, calcium, vitamin D, and phosphorus are essential for bone mineralization, while protein and amino acids provide the building blocks for bone tissue[27]. In addition, certain micronutrients such as magnesium, zinc, and copper are important cofactors in bone formation [28]. Anti-catabolic effects can be achieved through the intake of nutrients that inhibit bone resorption. For example, vitamin K has been shown to reduce the activity of osteoclasts, the cells responsible for bone resorption [29]. Similarly, certain phytoestrogens found in soy and other plants have been shown to inhibit osteoclast activity and promote bone formation [30]. Conversely, excessive intake of caffeine, alcohol, and sodium can lead to increased calcium excretion and bone loss [31].

Although there have been some investigations into the correlation between dietary micronutrients and osteoporosis, limited research has been conducted on the association between iron intake and osteoporosis, particularly in healthy adults. A U-shaped relationship between iron intake and osteoporosis, which is consistent with previous animal studies [32]. Population studies have discovered that excessive iron intake or iron metabolism-related diseases (e.g., hemochromatosis) can lead to reduced BMD [19, 33]. Iron deficiency anemia is positively related to BMD and an increased risk of osteoporosis and fractures [34]. Our research demonstrates that adequate iron intake is linked to a lower risk of osteoporosis in the healthy population. Even in those who have already had a fragility fracture, osteoporosis may be preventable and treated, and the moderate physical activity can significantly lower the risk of fracture and refracture [35]. Early screening, identification of populations at high risk for osteopenia, and prompt preventive action are all part of good public health practice. Our research indicates that increasing dietary iron intake is a useful strategy, but caution should be exercised to avoid excessive consuming.

The results of our study hold significant implications for public health. While the existing literature on dietary iron consumption in the general population remains limited, the relationship between iron levels in the blood and bone mineral density (BMD) has been extensively investigated. The association between serum ferritin and BMD in healthy individuals has yielded controversial findings. For example, one study found a positive correlation between serum ferritin and BMD in elderly men, but not women [36], while another study found an inverse correlation between BMD and either ferritin saturation or transferring in women over the age of 45, but not men [37]. In contrast to males, women’s dietary iron consumption was associated with osteoporosis/osteopenia at certain levels, according to our study. Women appear to be more susceptible to osteoporosis/osteopenia than males; for instance, osteoporosis is thought to afflict one-third of postmenopausal women globally, and women with osteopenia have a 1.8-fold higher chance of breaking a bone than do healthy women [38, 39]. To more accurately identify postmenopausal women with low BMD and to provide targeted recommendations for increasing dietary iron intake to replenish the iron loss in the body and reduce the risk of osteoporosis, additional biomarkers are necessary.

There are several mechanisms that could explain the association between iron intake and osteoporosis/osteopenia. Iron deficiency can negatively affect bone health by impairing bone, protein production and vitamin D metabolism. Iron is involved in the activation of the cytochrome P450 family and the catalysis of prolyl-4-hydroxylase and lysyl-hydroxylase, which are necessary for collagen synthesis [40, 41]. Collagen makes up approximately 90% of the protein in bone tissue, and hydroxylation of proline in pro-collagen is crucial for its synthesis [42–44]. Additionally, vitamin D plays a crucial role in maintaining bone health by enhancing calcium absorption in the stomach and maintaining the equilibrium of serum calcium and phosphate concentrations. The cytochrome P450 family is responsible for mediating and regulating these actions [45, 46]. Iron overload, on the other hand, has been found to promote osteoclast differentiation while impairing osteoblast proliferation and differentiation, thus leading to a decline in bone density [13, 15, 47]. In addition, the process of bone remodeling involves a close communication between osteoclasts and osteoblasts, which is regulated by intricate autocrine and paracrine mechanisms involving various regulatory proteins [48]. Previous mouse model suggested that iron overload has the potential to adversely affect the bone marrow microenvironment, leading to a decrease in both the quantity and quality of mesenchymal stem cells [49]. Another population study demonstrated that excessive iron accumulation can harm hematopoiesis by damaging both hematopoietic cells and the microenvironment in which they function. This process is facilitated by signaling proteins that are associated with ROS [50]. The above evidence provides important proof of the role of dietary iron intake in bone development and remodeling.

There were some strengths in the current study. Firstly, this study comprehensively assessed dietary iron intake. Firstly, our study comprehensively assessed dietary iron intake, which was estimated using the Food and Nutrient Database for Dietary Studies published by the United States Department of Agriculture. This database provides a reliable source of dietary intake data for the US population, and allowed us to evaluate the associations between iron intake and osteoporosis in a more precise and accurate manner. Secondly, the large sample size of our national study provided sufficient power to perform subgroup analyses, and allowed us to explore the potential differences in the association between iron intake and osteoporosis across different subpopulations. Thirdly, our study included participants over 20 years old, which is more applicable to health populations, compared with the research that focuses only on the elderly and postmenopausal women.

However, our study also has certain limitations that should be taken into consideration when interpreting our results. Firstly, the cross-sectional study design restricts our ability to establish causal associations between dietary iron intake and osteoporosis. Secondly, the dietary iron is the only micronutrient we included in all dietary intakes. It may be difficult to examine the synergistic effects of many nutrients. Thirdly, some covariates were self-reported, which might introduce recall error and affect the accuracy of our results. Finally, some potential covariates, such as genetic factors, Vitamin D, and blood iron levels, were not included in this study, which may affect the interpretation of our findings. Further longitudinal research is needed to confirm and extend our findings, and to address these limitations.

## Conclusion

In summary, moderate increases in dietary iron intake without overconsumption were substantially associated with a lower risk of osteopenia/osteoporosis in women. This study highlights the essential role of dietary iron intake in osteoporosis among women. To increase or maintain bone mass and reduce the risk of osteoporosis, public health and clinical interventions should take into account moderate increasing dietary iron intake without overconsumption as an important strategy for the individual and population levels.

## Data Availability

The data presented in this study are from publicly available data in NHANES (website: https://www.cdc.gov/nchs/nhanes/about_nhanes.htm).
